# The Full Mediating Role of Loneliness on the Relationship Between Social Support and Depression Among Rural Family Caregivers of Persons With Severe Mental Illness

**DOI:** 10.3389/fpubh.2021.729147

**Published:** 2021-10-28

**Authors:** Baiyang Zhang, Xin Lv, Mutian Qiao, Danping Liu

**Affiliations:** West China School of Public Health and West China Fourth Hospital, Sichuan University, Chengdu, China

**Keywords:** social support, loneliness, depression, caregivers, severe mental illness (SMI)

## Abstract

**Objective:** Depression is a common and overwhelming psychiatric disorder among family caregivers of persons with severe mental illness (SMI). The interrelationships among social support, loneliness, and depression, especially among this relatively vulnerable group, are poorly understood. The aim of the present study was to test the hypothesis that the social support contributes to the alleviation of depression, through its effect on reducing loneliness.

**Methods:** A survey of 256 rural family caregivers of persons with SMI was conducted between December 2017 and May 2018 in Chengdu City, Sichuan Province, China. Social support, loneliness and depression were measured. A series of multiple linear regression models and bootstrapping procedure were performed to examine the mediating effects of loneliness on the association between social support as well as its components and depression.

**Results:** The proportion of family caregivers of persons with SMI who reported significant depressive symptoms was 53.5%. Loneliness fully mediated the negative association between social support and depression. As to three components of social support, subjective support and objective support only had indirect associations with depression mediated by loneliness, while support utilization had both direct and indirect relationships with depression.

**Conclusion:** The current study highlighted that social support and its three components may acted as protective factors by decreasing the feelings of loneliness, which created a beneficial effect on depression among family caregivers of persons with SMI.

## Introduction

Severe mental illness (SMI) is commonly defined as a series of mental disorders that are persistent and disabling in nature, including schizophrenia, schizoaffective disorder, bipolar disorder, and other psychoses. In China, the total number of persons with SMI exceeded 16 million nationwide (1). Over 90% of persons with mental disorders are in a stable or basically stable condition, therefore they are often proposed to receive long-term rehabilitation treatment in the community and depend on their family members for care provision (2). It is well-documented that mental illness influences not only the persons diagnosed but also the caregivers who care for the sufferers (3–6). Depression is one of the common and overwhelming psychiatric disorder among caregivers of mentally ill persons (7, 8). The depressive symptoms among caregivers of mentally ill persons may arise from the disturbance in routine activities, social interaction, leisure time, and jobs resulted from the burdensome caregiving tasks. State of depression not only causes harm to the caregivers' overall health, but also may reduce their ability to provide care as well as the quality of care delivered to recipients (9). Moreover, depression further depletes the caregiver's own resources, increasing the care costs of both care provider and care recipient (10), and consequently, adding overall burden on patients' families. Therefore, identifying the factors of depression and exploring the underlying mechanisms among caregivers of persons with SMI warrants more attention.

Loneliness refers to a debilitating psychological condition characterized by a deep sense of emptiness, worthlessness, lack of control, and personal threat (11). Unlike the concept of living alone and social isolation, all of which indicate objective physical separation from others, loneliness is more inclined to subjective emotional status of being alone, separated, or apart from other people. Loneliness has been found to be a predictor of mental disorders, cognitive impairment, worse motor function, frailty, and even higher mortality (12–15). Due to consistent and long-term caregiving, caregivers of persons with SMI are more possible to remain isolated in homes with few social contact and social engagement (6), which may contribute to their sense of loneliness. The association between loneliness and depression has been identified among caregivers of persons with dementia (16), Alzheimer's disease (17) and chronic obstructive pulmonary disease (18). Nevertheless, there has been a paucity of research on the loneliness and its association with depression among the population of caregivers of persons with SMI.

Social support generally refers to the social resources that persons perceive to be available or that are provided to them (19). Derajew et al. found that social support was significantly related to depression among caregivers of persons with SMI (10). Song et al. indicated that inadequate social support was the most powerful predictor of depressive symptom among caregivers of mentally ill individuals (20). Houtjes et al. held the view that both the larger size of social network and lower degree of loneliness were vital predictors of the depression remission among older adults in Netherlands (21). In addition, the association between higher social support and fewer loneliness has been documented among the population of Chinese older adults (22–24) and caregivers of patients with cancer (25). Abella et al. further claimed that having a small social network was associated with loneliness, in particular among depressed subjects (26). Social support can fill the gap between the social network and the need for social contact, thereby alleviating loneliness.

As mentioned above, the associations between social support, loneliness, and depression, were well-documented; however, the interrelationships among these three variables, especially among the population of family caregivers of persons with SMI, is poorly understood. A study focusing on internet addicts demonstrated the partial mediating effect of loneliness on the association between social support and depression (27). In addition, similar results were found in the population of Chinese migrant children (28). Accordingly, the present study hypothesized that loneliness may also be one pathway through which social support may affect depression among caregivers of persons with SMI; and to our best knowledge, evidence is currently in a blank state. Moreover, prior researches concerning specific component of social support suggested that both overall social support (assessed by Social Support Rating Scale [SSRS]) and its three dimensions (i.e., subjective support, objective support, and support utilization) were negatively related to loneliness (23, 24) and depressive symptoms (29). Nevertheless, it remained unknown whether each social support dimension may act as different roles in the abovementioned hypothetical mediating framework. Hence, to fill the research gap, different components of social support were considered in the process of establishing mediation testing models.

Compared with the delivery of public care in urban areas, rural care systems have limited funding and face challenges in terms of the availability of qualified service providers (30). As a result, the care for individuals with SMI in rural areas may be more dependent on family caregivers. A prior research conducted in Nepal has shown that depression of rural caregivers of persons with SMI was more serious than that of caregivers in urban areas (31). Therefore, the current study focused on the family caregivers of persons with SMI in rural areas. The aims of the current study were to evaluating the interrelationships among social support, loneliness, and depression, as well as testing the mediating effect of loneliness on the association between social support and depression, in order to provide reference for improving psychological status of the relatively vulnerable groups, family caregivers of persons with SMI in rural areas.

## Materials and Methods

### Participants and Procedures

A cross-sectional study was conducted in Chengdu City, Sichuan Province, China between December 2017 and May 2018. To obtain participants, a rural district of this city was randomly selected as the survey area, and then ten townships were randomly selected in this rural district. According to the systematic sampling method, 30 registered persons with SMI in each township hospital were randomly selected and 300 family caregivers were obtained. Inclusion criteria of family caregivers were: family member of person with SMI, aging 18 and above, primary caregiver to the care recipient during the last year. After signing written informed consent, caregivers were interviewed face-to-face by professionally trained investigators. Of 300 eligible caregivers voluntary to participate, 256 completed data for final analysis, with an effective response rate of 85.3%. The study received approval from ethics committee of Sichuan University.

### Measures

Social support was assessed using the Social Support Rating Scale (SSRS), which was a self-report measure consisting of 10 items, grouped into three sub-scales: subjective support, objective support and support utilization (32). Subjective support denoted the perceived support network that an individual can count on. Objective support measured the actual or visible support an individual received. Support utilization reflected the utilizing degree of all support one can turn to. Item scores of were simply added up, generating three sub-scales score range of 8–32, 1–22, and 3–12, respectively. Total scale scores are the sum of the three sub-scales and range from 12 to 66, with a higher score standing for higher social support. The social support of respondents can be classified into low social support (scores of 12–22), moderate support (scores of 23–44), and high support (scores of 45–66). In this study, the Cronbach's α of the whole scale was 0.825.

Loneliness was measured using the UCLA Loneliness scale, which contained 20 items (33). Answers to the each UCLA item are given on a 4-point scale from 1 (never) to 4 (always). Total scores is calculated by summing up the score of each item, ranging from 20 to 80, with higher scores indicating higher loneliness. The cut-offs in using the UCLA scores are as follows: <48 indicating no/low loneliness, 48–63 indicating moderate loneliness, and more than 63 indicating high loneliness (11). The scale exhibited good internal consistency in the current study, α = 0.847.

Depression was operationalized with the Chinese version of the ten-item Center for Epidemiologic Studies Depression Scale (CESD-10), which reflected the respondents' depressive symptoms experienced over the previous week (34). The CESD-10 consists of 10 questions and each has four-point answers with a common stem, from 0 (experienced rarely or none of the time) to 3 (experienced most or all of the time). Respective answers (in a question) are scored in ascending order (questions 5 and 8 scored in reverse order). The total score of the ten items ranges from 0 to 30, with a higher score indicating higher possibility of depression. Individual with a score of 10 or higher is considered to have significant depressive symptoms (35). In this study, the scale had a Cronbach's alpha of α = 0.786.

The covariates included characteristics of family caregivers and persons with SMI for whom they cared, ruling out the potential confounding bias in examining mediating frameworks. Characteristics of family caregivers of included gender (men or women); age (<45, 45–59, ≥60 years); education level (no formal education, primary school, middle school, high school and above); marital status (married have spouses, divorced/widowed/unmarried); employment status (employed, unemployed, retired); annual personal income (<750$, 750–1499$, ≥1500$); chronic condition (yes or no); relationship with the care recipient (spouse, parents, other). Characteristics of persons with SMI included age (<45, 45–59, ≥60 years); medical insurance (yes or no); other chronic disease (yes or no) (e.g., hypertension, diabetes, chronic obstructive pulmonary disease, chronic gastric disease).

### Statistical Analysis

Descriptive statistics have been used to describe the sample, including means and standard deviations or frequencies and percentages. Pearson correlation coefficients were calculated to examine the correlated relationships among social support, loneliness and depression.

**Figure 1:**
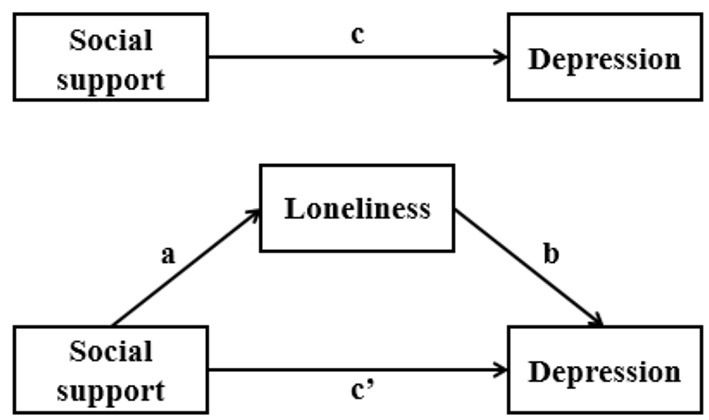
Hypothetical model of the interrelationships between social support, loneliness and depression.

According to Baron and Kenny (36), to establish mediating relationship, the following conditions are required: ^①^ The independent variable predicts the mediator variable and the dependent variable; ^②^ the mediator variable predicts the dependent variable; ^③^ after adjustment of the mediator variable, the significant impact of the independent variable on the dependent variable turns into non-significant (full or perfect mediation) or weaker (partial mediation). Therefore, a series of linear regression (Figure 1) were performed based on the abovementioned content to test the hypothetical mediating interrelationships. Firstly, the association between social support and depression (Path c) was examined, without considering the mediator variable loneliness. Next, the significance of the association between social support and loneliness was assessed (Path a). In the final step, both social support and potential mediator loneliness were entered simultaneously as predictors of depression (Path b and Path c'). In order to account for potential confounding bias, these regression models were conducted with adjustment of covariates. In addition, variance inflation factors and tolerances were assessed to verify that multicollinearity did not distort regression parameters.

As to estimating the size and significance of the mediation effect, Hayes' PROCESS macro model 4 (37) with the bootstrapping procedure were used. Bootstrapping was a non-parametric resampling procedure, which made no assumptions about the sampling distribution of the statistics, and was recommended due to its robust nature and ability in effect-size estimation and hypothesis testing (38). Typically, in abovementioned steps strategy, the indirect effect is the difference between the total and the direct effects of independent variable on dependent variable: ab = c-c'. The macro provided a bootstrap estimate of the indirect effect ab and confidence intervals for population value of ab. We performed 5,000 bootstrap resamples to calculate the bias-corrected 95% confidence interval of the indirect effect of social support on depression. The mediation effect was considered to be significant if the confidence interval did not include zero. The same procedure was conducted to analyze three dimensions of social support to determine different component's effects. Statistical analyses were conducted using SPSS 21.0 and the PROCESS macro for SPSS and a significance level of 0.05 was used throughout.

## Results

### Descriptive Statistics

Table 1 displays the descriptive statistics results of study sample. The proportion of women was slightly higher than men (54.3 vs. 45.7%). Nearly a half of the caregivers were aged <60 years. The majority of caregivers had an education level below high school (88.3%), and were married have spouses (81.6%). The proportion of caregivers who were unemployed was 33.6%. More than half of the caregivers had an annual personal income <1,500$ (58.6%) and 34.4% of the caregivers had chronic condition. As to the relationship with the care recipient, 31.6% of the caregivers were spouse while 39.5% of the caregivers were parents. The mean score for social support was 32.1 ± 7.7, and the proportion of family caregivers who had high level of social support was just 5.9%. The mean scores for subjective support, objective support and support utilization were 18.9 ± 5.2, 7.7 ± 2.3, and 5.6 ± 2.2, respectively. The mean score for loneliness was 45.3 ± 8.8, and more than half (54.7%) of family caregivers experienced moderate or high loneliness. The mean score for depression was 10.0 ± 5.3, and 53.5% of family caregivers reported significant depressive symptoms. As to persons with SMI for whom the family caregivers cared, 29.3% of them were aged ≥60 years. The majority of persons with SMI were covered by medical insurance (96.5%) and did not suffer from other chronic disease (80.1%).

**Table 1:** Descriptive statistics of characteristics for the study sample and their care recipients.

**Characteristics**	***N* (%), Mean ± SD**
**Family caregivers**	
**Gender**	
Men	117 (45.7)
Women	139 (54.3)
**Age (years)**	
<45	36 (14.1)
45–59	90 (35.2)
≥60	130 (50.8)
**Education level**	
No formal education	51 (19.9)
Primary school	106 (41.4)
Middle school	69 (27.0)
High school and above	30 (11.7)
**Marital status**	
Married have spouses	209 (81.6)
Divorced, widowed, or unmarried	47 (18.4)
**Employment status**	
Employed	125 (48.8)
Unemployed	86 (33.6)
Retired	45 (17.6)
**Annual personal income, $**	
<750	116 (45.3)
750–1,499	34 (13.3)
≥1,500	106 (41.4)
**Chronic condition**	
No	168 (65.6)
Yes	88 (34.4)
**Relationship with the care recipient**	
Spouse	81 (31.6)
Parents	101 (39.5)
Other	74 (28.9)
Social support	32.1 ± 7.7
Low social support	24 (9.4)
Moderate support	217 (84.7)
High support	15 (5.9)
Subjective support	18.9 ± 5.2
Objective support	7.7 ± 2.3
Support utilization	5.6 ± 2.2
Loneliness	45.3 ± 8.8
No/low loneliness	116 (45.3)
Moderate/high loneliness	140 (54.7)
Depression	10.0 ± 5.3
Had not significant depressive symptoms	119 (46.5)
Had significant depressive symptoms	137 (53.5)
**Persons with SMI**	
**Age (years)**	
<45	84 (32.8)
45–59	97 (37.9)
≥60	75 (29.3)
**Medical insurance**	
Yes	247 (96.5)
No	9 (3.5)
**Other chronic diseases**	
Yes	51 (19.9)
No	205 (80.1)

### Correlations Among Study Variables

Table 2 presents bivariate inter-correlations among study variables. As expected, social support and its three components were significantly negatively correlated with loneliness and depression. Loneliness and depression were positively correlated.

**Table 2:** Correlations among social support, loneliness, and depression.

	**Social**	**Subjective**	**Objective**	**Support**	**Loneliness**
	**support**	**support**	**support**	**utilization**	
Loneliness	−0.460^***^	−0.425^***^	−0.218^***^	−0.377^***^	
Depression	−0.300^***^	−0.263^***^	−0.154^*^	−0.267^***^	0.475^***^

* *p < 0.05*,

*** *p < 0.001*.

### Results of Mediation Analysis

Table 3 tabulates the results of the regression analyses examining the mediation hypothesis. The first step assessed whether social support was related to depression. The unstandardized regression coefficient for path c turned out to be statistically significant (β = −0.16, *p* < 0.001), indicating that a one-point increase in the social support score was associated with a decrease of 0.16 score in depression, with adjustment of caregiver characteristics. The second step examined whether social support predicted potential mediator among the study samples. The association between social support and loneliness (path a, β = −0.47, *p* < 0.001) was also found to be significant, suggesting that as social support grew, the loneliness decreased. The third step explored the direct effects of social support and loneliness on depression. Loneliness was positively associated with depression (path b, β = 0.27, *p* < 0.001). But social support was no longer a significant predictor of depression (path c', β = −0.04, *p* > 0.05), indicating that loneliness fully mediated the association between social support and depression and the study hypothesis was verified.

**Table 3:** Results of the regression models testing the mediation hypothesis.

**Variable**	**Total effect of social**		**Direct effect of social**		**Direct effects of social support on**	
	**support on depression**		**support on loneliness**		**depression and loneliness on depression**	
	**Path c**		**Path a**		**Path c' and Path b**	
	**B**	**SE**	**B**	**SE**	**B**	**SE**
Social support	−0.16^***^	0.04	−0.47^***^	0.07	−0.04	0.04
Loneliness	-	-	-	-	0.27^***^	0.04
Covariates Family caregivers						
Gender						
Men (ref)						
Women	2.17^**^	0.68	1.11	1.05	1.86^**^	0.62
Age (years)						
<45 (ref)						
45–59	0.22	1.05	1.63	1.62	−0.23	0.96
≥60	−1.51	1.18	2.98	1.83	−2.32^*^	1.08
Education						
No formal education (ref)						
Primary school	−2.28^*^	0.91	0.22	1.41	−2.34^**^	0.83
Middle school	−3.34^**^	1.04	−0.18	1.60	−3.29^**^	0.94
High school and above	−3.33^*^	1.32	1.28	2.05	−3.67^**^	1.20
Marital status						
Married have spouses (ref)						
Divorced, widowed, or unmarried	−0.39	0.91	−1.26	1.41	−0.04	0.83
Employment status						
Employed (ref)						
Unemployed	1.10	0.76	−1.83	1.17	1.59^*^	0.69
Retired	1.55	1.04	−0.56	1.61	1.70	0.95
Annual personal income, $						
<750 (ref)						
750–1,499	−1.08	0.99	−2.10	1.53	−0.51	0.91
≥1,500	−1.63^*^	0.72	−3.53^**^	1.11	−0.67	0.67
Chronic condition						
No (ref)						
Yes	−0.16	0.72	−1.12	1.11	0.15	0.65
Relationship with the care recipient						
Spouse (ref)						
Parents	−0.84	1.03	−1.31	1.59	−0.48	0.94
Other	−1.16	0.90	0.96	1.39	−1.42	0.82
Persons with SMI						
Age						
<45 (ref)						
45–59	−0.41	0.89	1.31	1.37	−0.76	0.81
≥60	−0.93	1.08	−0.96	1.67	−0.67	0.98
Medical insurance						
No (ref)						
Yes	1.00	1.75	7.57^**^	2.71	−1.06	1.62
Other chronic disease						
No (ref)						
Yes	−0.19	0.81	1.87	1.25	−0.70	0.74
Constant	26.12^***^	3.85	51.11^***^	5.95	9.73^**^	3.44
R^2^	0.23		0.32		0.36	
F	3.67^***^		5.74^***^		6.73^***^	

* *p < 0.05*,

** *p < 0.01*,

*** *p <0.001*.

Table 4 displays the size and significance results of direct effects and indirect effects of social support and its three dimensions on depression. The association between social support and depression was fully mediated by loneliness, with an indirect effect of −0.13 and a 95% confidence interval of −0.18 to −0.08. As to the three dimensions, the relationship between subjective support, objective support and depression were also fully mediated by loneliness; while both the direct and indirect association between support utilization and depression were significant, indicating partial mediation interrelationships.

**Table 4:** Direct and indirect effects of social support and its dimensions on depression.

**Effect**	**B**	**SE**	**95%CI**	
			**Lower**	**Upper**
**Social support on depression**				
Direct effect	−0.04	0.04	−0.12	0.05
Indirect effect	−0.13	0.03	−0.18	−0.08
**Subjective support on depression**				
Direct effect	−0.01	0.06	−0.14	0.11
Indirect effect	−0.18	0.04	−0.26	−0.11
**Osupport on depression**				
Direct effect	−0.02	0.14	−0.29	0.25
Indirect effect	−0.18	0.08	−0.36	−0.02
**Objective support on depression**				
Direct effect	−0.31	0.14	−0.59	−0.03
Indirect effect	−0.33	0.08	−0.51	−0.18

## Discussion

Family caregivers are fundamental in giving care to persons diagnosed with SMI. Caring for the SMI individuals is a long-term process that needs caregivers to invest endless time and effort, which may contribute to their psychological problems (35). Depression is a common adverse health consequence among family caregivers of persons with SMI and it affects family caregiver's day to day life (10). The current study examined the interrelationships among social support, loneliness, depression among family caregivers of persons with SMI and made the first attempt in exploring the heterogeneous effects of different social support components in the hypothetical framework. Results suggested the applicability of the hypothetical mediation model in determining depressive symptoms among family caregivers of persons with SMI.

In the current study, more than half (53.5%) of the family caregivers of persons with SMI had significant depressive symptoms, which was higher than that reported in Ethiopia (19%) (10), Sri Lanka (37.5%) (39), California USA (40%) (8), and Nepal (42.5%) (31). Such variation may be attributed to different culture settings. In the Chinese setting, especially in rural areas, the traditional aetiological beliefs about mental illnesses are still exerting influence, intensifying the stigma focused on persons with SMI and their family members (40) and then increasing caregivers' risks of developing depression. In addition, due to cultural factors, Chinese caregivers may have a stronger sense of obligation to provide care for their relatives than those in other countries (41). That is, for some caregivers, providing care was not of altruistic intention but out of duty. In this context, Chinese caregivers perceived lower caregiver esteem, which might lead to subjective burden and poor psychological status (41). Moreover, the depression prevalence of the study sample was also higher than that of caregivers of SMI persons in a rural area in central China (45.4%) (35). One possible explanation is that mental health service in the relatively underdeveloped southwest region is inferior to those in the central region of China (42). This situation may prevent persons with SMI from getting timely and effective treatment, thus increasing the intensity of caregiving, which may contributed to caregivers' depression (43).

Results of the study showed that the proportion of caregivers who reported moderate or high loneliness was 54.7%. Important life transitions that induce changes in one's existing or expected social interactions may precipitate the onset of loneliness (44). Family caregivers are generally informal, without previous care experience, thus taking on the caregiving burden often constitutes such a major life transition, resulting in their sense of loneliness. Consistent with previous literatures (16–18), the current study illustrated the positive association between loneliness and depression.

The findings in this study showed that only 5.9% of the caregivers reported high level of social support, indicating the social support of this population was rather poor. Caring for an individual with SMI may handicap many important social roles of caregivers. Consistent and long-term care task led to caregivers' inability to work outside of the home, communicate with others, freely manage their time and choice of space (44), and then resulted in their inaccessibility to social networks.

Our results supported the hypothesis that social support was negatively associated depression, with this relationship being fully mediated by loneliness. That is, social support acted as a protective factor by decreasing the feelings of loneliness, which created a beneficial effect on depression among family caregivers of persons with SMI. Nevertheless, previous studies focusing on Chinese internet addicts and migrant children demonstrated partial mediating frameworks (27, 28). The possible explanation of this discrepancy might be that the relatively special identity of family caregivers of persons with SMI placed them at a disadvantage in loneliness management. SMI individuals were usually considered to be impulsive, disruptive, violent and a source of unpredictable risks, thus the public tended to avoid contact with and isolated them (40, 45). This kind of discrimination attached to mental illness was also conferred upon caregivers (46), which limited the opportunities of contacting and sharing and kept them away from effective loneliness management. Hence, the buffer effect of social support was likely to be more attributed to the relieving of loneliness among family caregivers of SMI individuals than other groups.

In accordance with total social support, both the association between subjective support and depression, objective support and depression, were fully mediated by loneliness. These two components were usually conceptualized as “perceived” (i.e., subjective) and “received” (i.e., objective) support in many other researches, both of which had been linked to depression, health-related quality of life and well-being (47, 48). In addition, a qualitative study has pointed out that loneliness was not only related to a loss of social relationships, but also to a lack of satisfaction with existing moments of social interaction (44). Therefore, it is recommended that both subjective and objective support be considered in health intervention programs targeting the family caregiver population, so as to relieve their sense of loneliness and ultimately do good to their depression management. Support utilization reflected the utilizing degree of all support one can turn to and its negative relationships with loneliness was found in the current study. Prior study suggested that encounters in seeking social support were seen to generate loneliness (44). Results of the study showed that, besides the path mediated by loneliness, support utilization had direct negative association with depression, indicating that under the established social support circumstances, improving the utilization degree of support might be more essential in relieving depression among caregivers of persons with SMI.

It's worth noting that a study focused on Chinese elderly from cadre's sanitariums demonstrated the mediating effect of social support on the association between loneliness and depression (19). We have tried to assessing the mediating role of social support based on our data, but results did not support this conclusion. The stationary lagged effect of social support on loneliness has been proposed in a prospective study (49). And compelling evidence was provided by a 5-year longitudinal study focusing on older adults in Chicago which demonstrated that loneliness predicted subsequent depressive symptoms deterioration, but not vice versa (50). In addition, conclusions of previous studies supported the theoretical rationality of our hypothesis (27, 28). Prior research has pointed out that a variable may play multiple roles in the impact mechanism of outcomes, that is, it could be a leading variable of other variables affecting outcomes, and also be a mediator of other variables affecting outcomes (51). Therefore, we believed that the conclusions of the two studies were complementary to each other and longitudinal studies were recommended to confirm the mediating framework.

The current study shed new light on the fully mediating effect of loneliness on the association between social support and depression among family caregivers of persons with SMI and extended the content for different social support components' role in mediating network. The clinical implication of our findings may be considerable. Interventions aimed at strengthening social support, with a specific focus on reducing feelings of loneliness, may be beneficial in the prevention of depressive symptoms among family caregivers of persons with SMI. There is a need for intervention with caregivers, families and the community as a whole to establish support groups, which could help to enhance social support and manage their loneliness. In addition to focusing on the establishment of objective social networks, attention should be also paid to the caregivers' demand of emotional support, such as being understood and respected, as well as the availability of support utilization, so as to maximize the effect of social support in relieving loneliness and depression.

The current study has several limitations. Firstly, the study's cross-sectional nature prohibits the inference of any causal relationships among the variables. Longitudinal studies should be conducted in the future to provide more definite information and make up for this defect. Second, this study has adopted self-reported measurements which can be subjective and may exist some self-reported bias. Third, this research was conducted in rural areas of Chengdu City, Sichuan Province, southwest China and caution should be exercised when extending these results to other regions.

## Conclusions

The current study provides useful insights into the mechanism of the benefit effect of social support on depression among family caregivers of persons with SMI. The results obtained extended the literature by demonstrating the full mediating role of loneliness on the link between social support and depression. Interventions targeting to improve social support should be prioritized to promote psychological well-being of family caregivers of persons with SMI.

## Data Availability Statement

The raw data supporting the conclusions of this article will be made available by the authors, without undue reservation.

## Ethics Statement

The studies involving human participants were reviewed and approved by Ethics Committee of Sichuan University. The patients/participants provided their written informed consent to participate in this study.

## Author Contributions

DL and BZ: conceptualization. DL: methodology. BZ and XL: software and formal analysis. BZ: writing—original draft preparation. BZ, XL, and MQ: investigation. BZ, XL, MQ, and DL: writing—review and editing. All authors contributed to the article and approved the submitted version.

## Conflict of Interest

The authors declare that the research was conducted in the absence of any commercial or financial relationships that could be construed as a potential conflict of interest.

## Publisher's Note

All claims expressed in this article are solely those of the authors and do not necessarily represent those of their affiliated organizations, or those of the publisher, the editors and the reviewers. Any product that may be evaluated in this article, or claim that may be made by its manufacturer, is not guaranteed or endorsed by the publisher.
